# Stimuli-responsive Prussian blue analogues†

**DOI:** 10.1039/d5tc01760b

**Published:** 2025-07-10

**Authors:** Hanna L. B. Boström, Yevheniia Kholina, Arkadiy Simonov

**Affiliations:** a Department of Chemistry, Stockholm University Svante Arrhenius väg 16C SE-106 91 Stockholm Sweden hanna.bostrom@su.se; b Wallenberg Initiative Materials Science for Sustainability, Department of Chemistry, Stockholm University SE-114 18 Stockholm Sweden; c Department of Materials, ETH Zürich Vladimir-Prelog-Weg 4 8093 Zürich Switzerland arkadiy.simonov@mat.ethz.ch

## Abstract

The metal–cyanide frameworks known as Prussian blue analogues display a suite of diverse and tuneable properties. In particular, they are often highly susceptible to modification by external stimuli, such as temperature, pressure, radiation, adsorbed guests, or electric/magnetic fields. This can lead to rare and intriguing phenomena—including humidity-induced magnetism, electrochromism, or light-switchable electronic bistability—which enable a wide range of potential applications. The present article briefly surveys the stimuli-responsive behaviour of Prussian blue analogues, including spin crossover, charge transfer, chromism, conductivity, magnetism, and strain. Throughout, areas with potential for future developments are highlighted.

## Abstract 2

We review the responses of Prussian blue analogues to light, pressure, magnetic/electric fields, temperature, and guest absorption.

## Graphical abstract

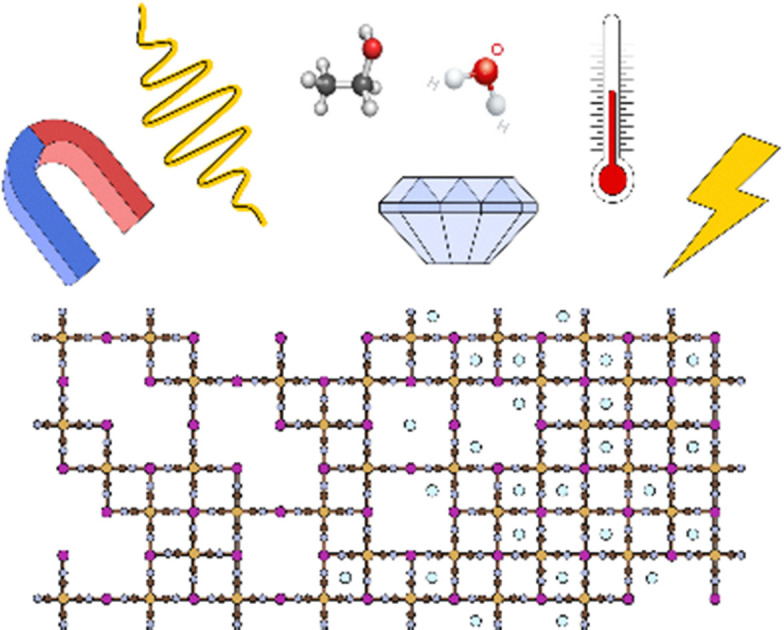

## 1 Introduction

Despite credited as the oldest known coordination polymers,^1,2^ Prussian blue analogues (PBAs) continue to attract research attention. As these compounds reside at the intersection of perovskites and metal–organic frameworks (MOFs), they combine the best of two worlds of materials. Like oxides, they can exhibit redox behaviour and metal–metal correlations, which leads to applications within electrochemistry and magnetism.^3–5^ But like MOFs, many PBAs are microporous with open metal sites (M-site), enabling catalysis^6,7^ and gas storage.^8,9^ A combination of microporosity and magnetic coupling is also possible, which opens up for rare phenomena such as humidity-induced magnetism.^10^ This multifunctionality sets PBAs apart from many other classes of materials and yields a broad spectrum of potential applications.

The versatility of PBAs is underpinned by the intriguing structural chemistry, which in turn stems from the stoichiometric and compositional flexibility of the crystal structure. PBAs consist of a rocksalt arrangement of (transition) metals M and M′, linearly joined by cyanide ions.^11,12^ At its simplest, this results in the formula MM′(CN)_6_. Due to the asymmetric crystal field strength of the cyanide ion, the M and M′ metals are in a high spin (HS) and low spin (LS) state, respectively. Consequently, these species are electronically inequivalent even if they correspond to the same element. If the charges of the transition metals fall short of +6, alkali ions (A) are inserted at the tetrahedral interstitial sites, or M′(CN)_6_ units removed, to achieve charge balance. This gives the formula A_*x*_M[M′(CN)_6_]_1−*y*_□_*y*_·*n*G, where the A-site occupancy (*x*) and the amount of M′-site vacancies/defects (*y*) stipulate the stoichiometry. G denotes a generic guest molecules, often water. By way of example, porous M^II^[M′^III^(CN)_6_]_2/3_ is obtained for *x* = 0 and *y* = 1/3. The defects/vacancies (*y*) arrange in a correlated manner—albeit without long-range order—which opens up for tuning of the pore structure.^13,14^ At the other end of the stoichiometric spectrum, perovskite-like, denser A_2_M^II^M′^II^(CN)_6_ form for *x* = 2 and *y* = 0. This compositional flexibility gives a large scope for property optimisation.

**Fig. 1:**
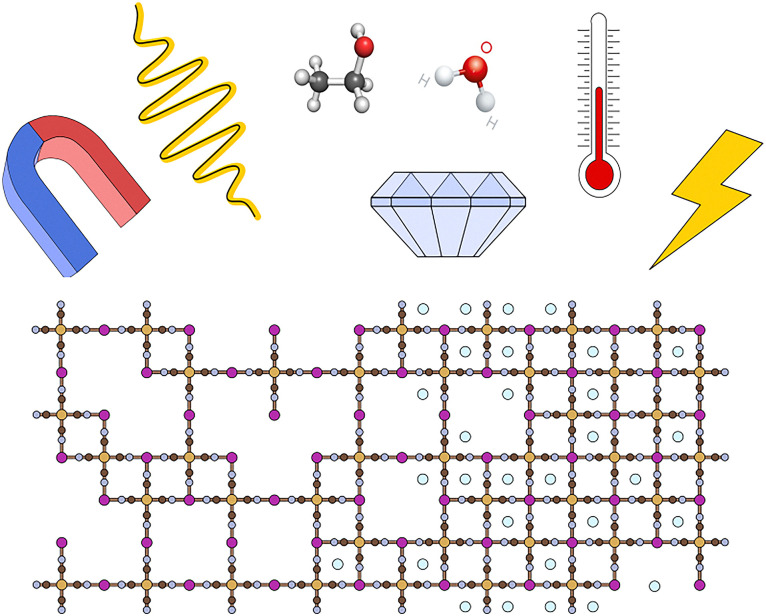
The variable stoichiometry of Prussian blue analogues and the stimuli considered in this study. A-site cations are shown in light blue and transition metals in yellow and purple.

A key feature of many PBAs is bistability, *i.e.* they can support two distinct optical/magnetic states. These states can be interconverted by an external stimulus, such as temperature, light, pressure or electric field [Fig. 1].^15^ By way of example, the first observation of metal–metal charge transfer in PBAs was reported in 1996, when red light was found to enhance the magnetisation and magnetic ordering temperature in K_0.14_Fe[Co(CN)_6_]_0.71_.^16^ This spurred extensive attention in the area of switchable PBAs and since then, numerous other stimuli and types of stimuli-responsive behaviour in this class of materials have been discovered. The tunability of PBAs and susceptibility to external stimuli are highly appealing for uses within *e.g.* memory storage,^17^ molecular switching,^15^ or sensing.^18^

The present review highlights the various types of stimuli-responsive behaviour found in PBAs. This is a vast field of research and, rather than providing a comprehensive account, we briefly discuss key examples of particular currency. References to more extensive reviews are given throughout. The manuscript commences by introducing spin crossover and metal–metal charge transfer: mechanisms which underlie several of the stimuli-responsive functionality discussed subsequently. A range of properties susceptible to external stimuli are then presented in turn, including colour, conductivity, magnetism, and strain. For each, the effects of temperature, pressure, radiation, electric/magnetic field, and solvent are outlined. When known, the structure–property relationships are discussed, referring to the effects of both the stoichiometry and composition of the PBAs on the relevant properties. The review concludes by discussing types of stimuli-responsiveness missing from the above presentation, highlighting avenues for further research.

## 2 Spin crossover

**Fig. 2:**
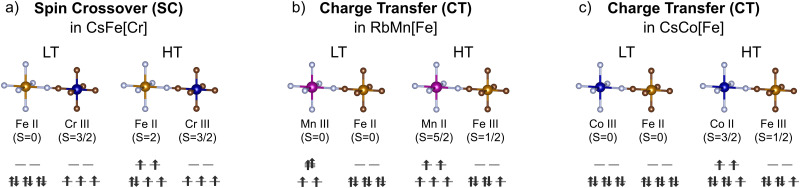
The electronic transitions and spin state changes during spin crossover and charge transfer in PBAs. (a) Spin crossover in CsFeCr(CN)_6_ and charge transfer in (b) RbMnFe(CN)_6_ and (c) CsCoFe(CN)_6_.

Spin crossover (SCO) involves the transition between the high and low spin states of a metal ion, often Fe^II^ ions coordinated by N-bearing ligands [Fig. 2]. To distinguish it from charge transfer, we here define spin crossover as a transition localised on one metal ion without changes in oxidation state. Abrupt transitions are often desired and this can be facilitated by high cooperativity between the SCO-active metal centres.^19^ In general, coordination polymers with rigid ligands are strongly cooperative,^19^ and hence, PBAs should be good candidates for SCO on account of their rigid cyanide linkers. Indeed, the archetypical SCO-active coordination polymers Hofmann complexes can be described as layered PBAs structures.

Although PBAs can be expected to exhibit favourably cooperative SCO properties, only one compound with thermal SCO is reported to date.^20^ CsFeCr(CN)_6_ shows thermal spin crossover around 225 K with a hysteresis loop of 27 K, whereas the related Fe[Cr(CN)_6_]_2/3_ remains in the high spin state upon cooling.^20^ The fact that vacancies inhibit SCO was attributed to the reduced crystal field around the Fe^II^ ion in Fe[Cr(CN)_6_]_2/3_, as 2 NC ligands are (on average) replaced by H_2_O ligands. While this study has been followed up by a plethora of theoretical studies,^21,22^ no exploration of related compositions for SCO behaviour appears to have been carried out to date.

The thermal SCO transition of CsFeCr(CN)_6_ is sensitive to compression and the hysteresis loop shifts to higher temperatures under the application of external pressure.^23^ Merely 1.7 kbar is sufficient to reversibly shift the transition above 270 K with presumed preservation of the hysteresis loop.^23^ Pressure-induced SCO was also reported in passing for Cr^II^[Cr^III^(CN)_6_]_2/3_ (critical pressure = 4 kbar), but few details were provided.^24^ Assuming that the stated composition for this compound is accurate, vacancies do not necessarily prevent pressure-induced SCO—unlike thermal SCO. The low pressures required are attractive for potential applications close to ambient conditions. Substantially higher transition pressure (∼0.8 GPa) was observed for the spin transition in FePt(CN)_6_, which is thermally inaccessible.^25,26^ The large difference in critical pressures between hexacyanochromates and -platinates could give tunability by means of solid solutions. Furthermore, exploration of the compositional space surrounding these two known SCO systems would be valuable.

An intriguing observation made for both CsFeCr(CN)_6_ and FePt(CN)_6_ is the interplay of the SCO transition with X-ray radiation.^25,27^ In the former, X-ray exposure can induce the H → LS transition both within and above the thermal hysteresis loop with preservation of crystallinity.^27^ For FePt(CN)_6_, X-ray exposure at pressures below the transition led to ∼80% conversion to the LS state on the timescale of minutes.^25^ The energy of the X-rays exceeds the energy difference between the HS and LS states and so the mechanism is not obvious, but postulated to involve photoionisation and de-excitation into the LS state.^27^ Further studies are required here, and the ability to measure accurate radiation doses—as commonly seen in macromolecular crystallography^28^—will be a useful asset. Apart from the X-ray effects, the photosensitivity of these transitions—using *e.g.* visible light—does not appear to have been studied to date.

Guest effects are rampant in SCO systems, and provide an attractive route towards sensors.^29^ Many mechanisms may underlie such behaviour, but the most intuitive is underpinned by the relative size of the guest: large guests will resist the strain associated with the SCO transition and favour the HS state.^29^ The role of guest for SCO has hardly been studied at all in PBAs, but compression of FePt(CN)_6_ in both penetrating and non-penetrating pressure-transmitting media (PTM) revealed no effect of the medium on the SCO transition.^25^ However, it is difficult to ascertain whether a PTM really has entered the pores or not. As the small pore volumes of PBA in comparison to *e.g.* MOFs or Hofmann complexes will restrict the number of suitable guests, understanding the role of vacancies in the SCO behaviour will be key. Furthermore, PBAs with vacancies possess open metal sites, and so guest coordination could provide another means towards modulating the spin states.

Altogether, SCO is relatively underexplored in PBAs, in particular in contrast to the large attention received by charge transfer transitions, outlined in the next section. With only 2–3 known SCO-active compositions, there are plenty of avenues for exploring new compounds with potential SCO behaviour, the sensitivity to various stimuli (temperature, pressure, and radiation), as well as the guest effects. While there is some understanding of the stoichiometric requirements for SCO—vacancies appear detrimental at least for thermal transitions—more research would be valuable in this area as well.

## 3 Metal–metal charge transfer

Metal–metal charge transfer (CT) in PBAs is a process where one transition metal acts as an electron donor and the other as an electron acceptor [Fig. 2]. The electron transfer event can be triggered by changes in temperature, light excitation, pressure, or electric field.^16,30–32^ Like SCO, CT is typically associated with altered bond lengths and leads to drastic changes in the magnetic and optical properties.^33^ So far, charge transfer has been observed in PBAs with a wide range of MM′-combinations, including MnFe, CoFe, FeOs, and CoOs.^15,34^ However, the most studied systems are A_*x*_Mn[Fe(CN)_6_]_1−*y*_ and A_*x*_Co[Fe(CN)_6_]_1−*y*_, and these will be the focus of this section.

In Rb_*x*_Mn[Fe(CN)_6_]_1−*y*_, switching between the high-temperature (HT) state Mn^II^Fe^III^ and a low temperature (LT) state Mn^III^Fe^II^ occurs in the temperature range 241–304 K with a very wide hysteresis loop.^33^ The electronic transition is accompanied by a structural cubic–tetragonal phase transition, driven by the Jahn–Teller distortion of Mn^III^ [Fig. 2]. Stoichiometry is key to the CT: both the transition temperature and the width of the hysteresis loops depend on the amount of vacancies, and the CT vanishes above a certain vacancy concentration.^35,36^ In addition to A = Rb, the Cs analogues also show CT transitions with substantial hysteresis,^37^ but studies involving the other alkali ions are scarce.

**Fig. 3:**
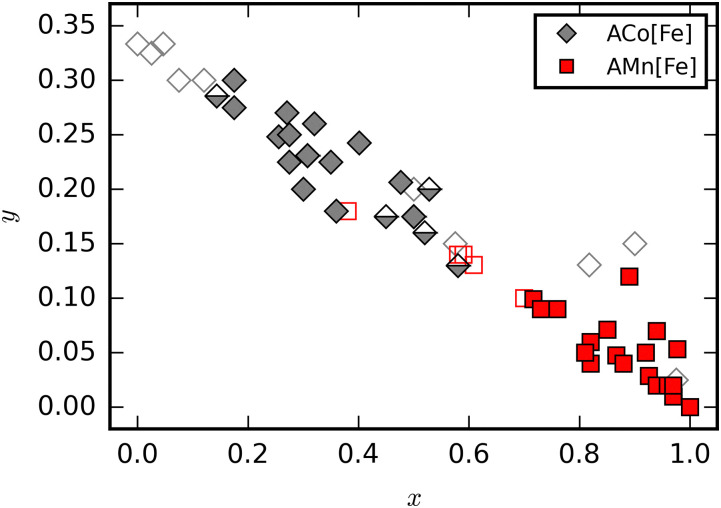
PBAs with formula A_*x*_Co[Fe(CN)_6_]_1−*y*_ (grey diamonds) and A_*x*_Mn[Fe(CN)_6_]_1−*y*_ (red squares) as a function of *x* and *y*. Filled (empty) symbols indicate thermally accessible (inaccessible) CT transitions. Half-filled symbols indicate that the CT transition can be induced by radiation but not thermally.

Charge transfer in A_*x*_Co[Fe(CN)_6_]_1−*y*_ proceeds without global symmetry changes. The two bistable states are the Fe^III^Co^II^ (HT) and Fe^II^Co^III^ (LT) electronic configurations [Fig. 2].^16^ The Co ion undergoes a SCO transition as a result of the CT, and the LT state is diamagnetic. Thermal charge transfer is typically induced at temperatures between 170 and 300 K,^15^ and again varies with the stoichiometry. However, in contrast to A_*x*_Mn[Fe(CN)_6_]_1−*y*_, vacancies facilitate CT for this family. This is explained on the basis of flexibility: the switching in A_*x*_Co[Fe(CN)_6_]_1−*y*_ involves local structural changes, such as changes to the metal–ligand bond lengths (Δ ∼ 0.2 Å). To absorb this dilatation and stabilise an excited state, flexibility is required. Hexacyanoferrate vacancies behave as relaxation points for the network strain and thereby facilitate the charge transfer.^38^ Thus, vacancies are essential for the CT transition in A_*x*_Co[Fe(CN)_6_]_1−*y*_ and so the compositional requirements for charge transfer are system dependent [Fig. 3].

Another way to induce charge transfer in either A_*x*_Co[Fe(CN)_6_]_1−*y*_ or A_*x*_Mn[Fe(CN)_6_]_1−*y*_ is to apply hydrostatic pressure.^31,39^ CT under compression can be observed even in compounds that do not switch thermally, *e.g.* highly defective K_0.03_Co[Fe(CN)_6_]_0.68_.^40^ Compression generally favours the LS state, which has a smaller volume. In systems with thermal CT, the application of pressure often shifts the transition to higher temperatures.^31,39^ Even small variations in applied pressure (kbars) can give considerable increases in transition temperatures, shifting them towards ambient temperatures.^31^ Furthermore, applied pressure allows access to room-temperature photomagnetic switching, as observed in K_0.03_Co[Fe(CN)_6_]_0.68_.^40^ The system undergoes CT at 2 GPa and irradiation by visible light at the same pressure reverses the process. This metastable excited state is stable for hours.^40^ Clearly, pressure is an important stimulus in the context of charge transfer transitions.

Photoinduction of the CT process is possible in both A_*x*_Co[Fe(CN)_6_]_1−*y*_ and A_*x*_Mn[Fe(CN)_6_]_1−*y*_.^16,41^ The former undergoes ferrimagnetic ordering below 16 K and the low-spin state can be transformed to the high-spin state under red or green light irradiation.^16^ Partial reversal is accessible *via* irradiation with blue light.^16^ Ultrafast studies show that the spin transition on Co occurs first, thus driving the electronic transfer.^42^ Likewise, the two electronic states of compounds in the A_*x*_Mn[Fe(CN)_6_]_1−*y*_ family can be interconverted by irradiation within the thermal hysteresis loop.^41,43^ The transition leads to a clear change in the magnetic properties and also changes the nonlinear optic response.^44^ In addition, CT can sometimes be induced in samples located at the edges of the stoichiometric window for thermal CT [Fig. 3].^15^ Light is a trigger for applications within information storage,^15^ and so the relative prevalence of photoinduced CT in PBAs may be of technological relevance.

Absorbed guest molecules can interplay with the CT in PBAs, although detailed studies are scarce. By way of example, interstitial water is a prerequisite for charge transfer to occur in Na_0.46_Co[Fe(CN)_6_]_0.78_·1.31H_2_O.^45^ This observation was attributed to a change in coordination geometry (octahedral to tetrahedral) of the Co^II^ ions following dehydration.^45^ This effect also underpins the thermochromism of Co^II^[Co^III^(CN)_6_]_2/3_ and will be discussed below.^46,47^ Although guests can have a substantial effect on electronic transitions in general,^29^ few additional studies on this phenomenon in PBAs have been carried out.

Lastly, charge transfer can be electrically induced in Rb_0.8_Mn[Fe(CN)_6_]_0.93_, by applying a voltage above 1.2 kV mm^−1^.^32^ The process can be repeated by resetting the material by heating. This is a very attractive scheme, since operation only requires two contacts; however more research is required for a solid fundamental understanding. For instance, it is unclear whether electric voltage can be used to also reverse the transition. Additionally, the underlying mechanism is poorly understood, especially the role of electrochemical reduction. Nevertheless, these initial results show promise and further studies are encouraged.

To summarise, charge transfer in PBAs in A_*x*_Co[Fe(CN)_6_]_1−*y*_ and A_*x*_Mn[Fe(CN)_6_]_1−*y*_ is relatively well studied. In both cases, the transition characteristics can be tuned by varying the stoichiometry, and possibly also the A-site metal.^48^ It is interesting that the compositional windows where the CT is thermally accessible differs strongly for the two families, but the underlying reasons do not appear to have been investigated. Both pressure and radiation can stimulate transitions outside this compositional window. Overall, the susceptibility to a range of stimuli is intriguing, as well as the emerging mechanistic insight into the electronic process.

## 4 Chromism

Colour is a striking property of PBAs; indeed the eponym Prussian blue, Fe^III^[Fe^II^(CN)_6_]_3/4_, is a well-known pigment featuring on several famous artworks.^49^ The vivid colour arises from intervalence charge transfer between the two transition metals and the optical properties thus depend on the composition. A wide range of colours are accessible by varying the transition metals, *e.g.* pink,^46^ yellow,^8^ purple,^45^ and green.^45^ The strong colours and their interplay with spin transitions form the basis for potential applications within *e.g.* optical sensing.

**Fig. 4:**
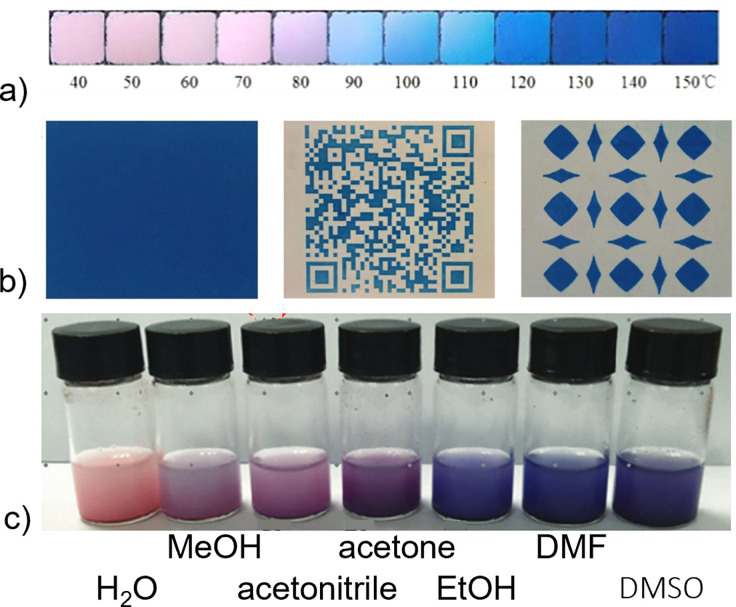
(a) Thermochromism in Co^II^[Co^III^(CN)_6_]_2/3_,^51^ reprinted from ref. 51 with permission. Copyright (2018) Elsevier. (b) Patterns printed on rewritable paper based on the photochromism of PB.^52^ Reprinted with permission from ref. 52. Copyright 2017 American Chemical Society. (c) Solvochromism in Co^II^[Co^III^(CN)_6_]_2/3_.^53^ Reproduced from ref. 53 with permission from the Royal Society of Chemistry.

Thermochromism is reported in a few PBAs, most notably in Co^II^[Co^III^(CN)_6_]_2/3_, where the coordination of the Co^II^ ion is key.^46^ At room temperature, Co^II^ is octahedrally coordinated by (on average) 4 NC ligands and 2 H_2_O molecules. The water evaporates on heating and the local coordination environment becomes more tetrahedral, leading to a colour change from pink to blue [Fig. 4(a)].^46^ The exact transition range can be tuned by the addition of citrate anions, which increase the binding energy of the water and thereby delay the thermochromic transition.^46^ In principle, changing the metals should allow a range of colours to be obtained, as evidenced by the brown–green thermochromism of a CoFe-based PBA.^50^ In addition to gradual thermochromism, both SCO and CT transitions can give abrupt colour changes.^33,45^

Many photoactive PBAs exhibit photochromism—light-induced colour changes—often as a result of charge transfer or photoreduction.^54–56^ For example, it has long been known that Prussian blue can be photoreduced by UV radiation to Prussian white, A_2_FeFe(CN)_6_.^57^ This can be undesirable in some cases, and *e.g.* leads to fading of artworks painted with Prussian blue-based paint.^49^ On a positive side, this photosensitivity has been exploited to create light-printable, rewritable paper based on nanoparticular Prussian blue and TiO_2_ [Fig. 4(b)].^52^ The rewritable paper was exposed to light through a photomask, allowing text and patterns to be printed. Oxidation in air gradually reverses the process, allowing for re-use.^52^ Another mechanism towards photochromism is radiation-induced electronic transitions, as outlined in previous sections.^36^ Altogether, photochromism is integral to PBAs, with considerable potential for exploitation in both technology and art.

Thanks to the porosity of defective PBAs, solvochromism and/or vapochromism is possible. This typically arises from the interaction of solvents with open metal sites, thereby modulating the crystal field. Indeed, the aforementioned thermochromism of Co^II^[Co^III^(CN)_6_]_2/3_ is based on a hydrochromic mechanism.^46^ Similar results are also known in analogous hexacyanochromates.^47^ Looking beyond water, organic solvents change the optical properties in these systems from pink to dark blue [Fig. 4(c)].^53^ Dark-field microscopy experiments show that not only the absorption spectra but also scattering spectra can be used for such detection.^58^

The redox-active nature of Fe-based PBAs leads to substantial scope for electrochromism. Indeed, the parent Prussian blue—Fe^III^[Fe^II^(CN)_6_]_3/4_—can be oxidised to Fe^III^Fe^III^(CN)_6_ (Prussian yellow) or reduced to A_2_Fe^II^Fe^II^(CN)_6_ (Prussian white); processes associated with obvious colour changes. This belies numerous applications, including electrochromic windows,^59^ electrochromic fingerprint detection,^60^ or biosensing of *e.g.* peroxides, urea and antioxidants.^61–63^ A range of wearable devices with biosensing functionality for real-time healthcare monitoring, largely based on Prussian blue, have also been developed.^18^ The introduction of transition metals other than Fe diversifies the range of accessible colours and enables *e.g.* multicolour displays.^64^ It follows that electrochromism is widely applicable, and is arguably the most mature type of chromism in PBAs for device integration.

To conclude this section, numerous forms of chromisms are known, many of which show great promise for applications. Accessing different colours is relatively easily performed by metal substitution and the switching is typically highly cyclable.^52,59,64^ Conversely, solvochromism in PBAs has attracted less interest for applications. Moreover, there are few studies directly investigating piezochromism in PBAs, although this is accessible *via* electronic transitions.

## 5 Conductivity

Prussian blue analogues show a range of conductive properties. First, many members of the family contain mobile alkali ions and thus are ion conductors, which has lead to their widespread use as cathode materials.^3,65^ Second, many hydrated PBAs show proton conductivity *via* a hopping (Grotthus) mechanism,^66^ and, lastly, some dry PBAS also show electronic conductivity.^67^

The conductivity can be readily studied as PBAs can be conveniently grown on interdigitated electrodes using *e.g.* electrochemical deposition, inkjet printing, or simple sequential dipping in precursor solutions.^68^ Both DC and AC measurement schemes have been successfully employed, though DC measurements involve simultaneous reduction of Prussian blue at the negatively charged electrode and oxidation at the positive one.^69^ As a brief aside, it is worth noting that an entire field of research is dedicated to using PBA-modified electrodes for H_2_O_2_ detection, which can be extended to various natural molecules when combined with oxidases.^70,71^

One of the earliest demonstrations of the sensing abilities of PBAs is humidity detection, exploiting the water sensitivity of the PBAs′ conductivity.^72^ The mechanism of this sensitivity is not only *via* hydrogen conduction, but also relies on the decrease of the ionic conductivity—up to 5 orders of magnitude—following water removal, as cation hydration is required for ionic mobility.^66,72,73^ In addition to water, PBA conductivity is also reported to be sensitive to vapour of methanol and dichloroethane.^73^

The conductivity of PBAs may be modified by direct electrochemical manipulation of the oxidation states. The potential of PBAs for resistive switching applications was demonstrated in electrodeposited Prussian blue layers, which showed bipolar switching behaviour with a 1000-fold change in conductivity.^74^ More recently, this concept has been significantly advanced using Li_*x*_Ru[Ru(CN)_6_]_1−*y*_.^17^ This system demonstrates reversible conductance switching over four orders of magnitude through electrochemically tunable oxidation states. Such memristive behaviour, with its ability to maintain multiple conductance states similar to biological synaptic weights, renders these materials promising for neuromorphic computing. The combination of precise conductance control through voltage pulses, excellent state retention, and relatively simple fabrication process addresses several key challenges in developing practical neuromorphic systems.^17^

The interplay between conductivity and other stimuli-responsive phenomena in PBAs is intriguing. By way of example, the electrical conductivity of Co[Cr(CN)_6_]_2/3_ and V[Cr(CN)_6_]_2/3_ shows sensitivity to magnetic phase transitions.^75^ While the effect is subtle, manifesting only as a small anomaly in the temperature-dependent conductivity at *T*_c_, the very existence of coupling between magnetic ordering and electrical transport is noteworthy. The effect is likely mediated by magnetostrictive distortions of the hydrogen bonding network below *T*_c_. Given the minor nature of these structural changes, the small magnitude of the response is unsurprising. Conversely, in Rb_0.76_Mn[Fe(CN)_6_]_0.91_, the CT transition produces surprisingly modest changes in electrical conductivity,^76^ despite the considerable volume change. Hence, the relationship between structural changes and electrical transport in PBAs is more nuanced than might be expected from simple geometric considerations. There are also claims of magnetoresistance effects during voltammetric scanning, though these observations rely primarily on indirect evidence correlating conductivity changes with inferred changes to the magnetic behaviour.^77^

The diverse conductive properties of PBAs, from ionic to electronic conductivity, coupled with their sensitivity to various stimuli, are attractive for a range of applications. Their ease of integration into devices through simple fabrication methods on electrodes further enhances their practical utility. However, several aspects of their conductive behaviour remain to be fully explored. In particular, photoinduced effects on conductivity and pressure-dependent conductivity are promising areas for future investigation.

## 6 Magnetism

The magnetic functionalities of PBAs have been well studied over many decades. In particular, the ease of metal substitution and Kahn's theoretical model have enabled the design of magnetic PBAS with ordering temperatures above ambient temperature.^5^ Many in-depth reviews about general magnetism in PBAs are available,^5,34,78^ whereas this section specifically focuses on unusual magnetic responses that may be induced by the various external stimuli.

**Fig. 5:**
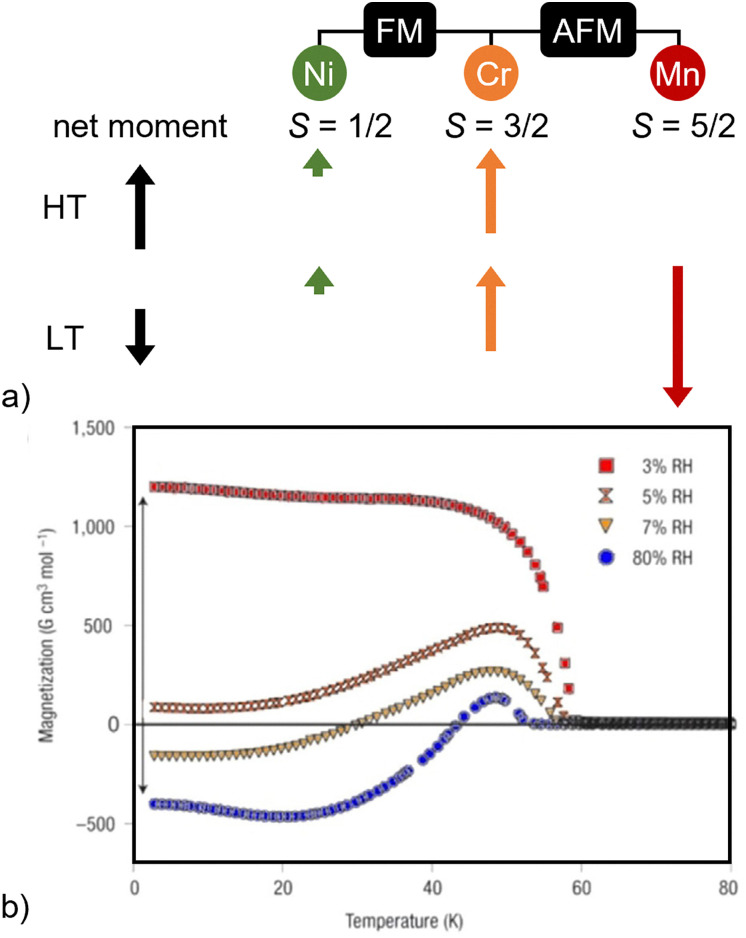
(a) The evolution of negative magnetisation in Ni_*x*_Mn_1−*x*_[Cr(CN)_6_]_2/3_. The magnetic moments of the different ions at high and low temperature (HT/LT) are illustrated. (b) The humidity-sensitive negative magnetisation of Co_0.41_Mn_0.59_[Cr(CN)_6_]_2/3_.^82^ Reproduced from ref. 82 with permission from Springer Nature.

Any magnetic order can of course be categorised as thermally induced magnetism, but PBAs can also show more counterintuitive magnetic behaviour in response to temperature changes. One example is negative magnetisation, which occurs in some mixed ferro-ferrimagnets, such as Cu_0.73_Mn_0.77_Fe(CN)_6_ or Ni_*x*_Mn_1−*x*_[Cr(CN)_6_]_2/3_ [Fig. 5(a)].^79,80^ Using the latter as an example, the Ni^II^ ion (*S* = 1/2) orders ferromagnetically with the Cr^III^ ion (*S* = 3/2) ion to give a positive magnetisation. Upon further cooling, Mn^II^ (*S* = 5/2) orders antiferromagnetically with the Ni–Cr pair and due to its larger magnetic moment, the result is a net negative magnetisation below 18 K.^79^ Even higher levels of complexity are achievable: a PBA with two compensation temperatures (*T*_comp_, *i.e.* sign changes of the magnetisation) has been designed by incorporating four transition metals with different magnetic moments.^81^

External pressure is an important tool in molecular magnetism,^83^ and PBAs exhibit plenty of interesting magnetic responses when compressed. For example, the mixed ferro-ferrimagnet Rb_0.64_Ni_0.31_Mn_0.87_Fe(CN)_6_ shows a pressure-induced magnetic pole inversion at only 0.2 kbar.^84^ This arises from enhanced ferromagnetic interactions (Ni–Fe) upon compression relative to the antiferromagnetic exchange (Mn–Fe).^84^ Similarly, pressure can drastically increase the magnetic ordering temperatures for ferrimagnetic PBAs.^85,86^ In addition, pressure-induced linkage isomerism of the cyanide ion has been observed in certain hexacyanochromates, which weakens the magnetic susceptibility.^87,88^ These examples showcase a diverse and composition-specific magnetic behaviour under pressure.

Photomagnetism is an important property of PBAs, with a bearing on both fundamental and applied research. Most known examples originate from the spin state changes caused by photoinduced charge transfer, including changes from paramagnetism to ferrimagnetism^89^ and photodemagnetisation [Section 3].^90^ Another intriguing effect is the photoinduced magnetic pole inversion in Fe_0.4_Mn_0.6_[Cr(CN)_6_]_2/3_,^91^ which shows negative magnetisation below *T*_comp_ = 19 K. Irradiation with blue light changes the sign of the negative magnetisation, whereas gentle heating returns the system to the initial state.^91^ This results from a photodemagnetisation of the Fe–Cr ferromagnetic substructure—where the mechanism is still unclear—whereas the Mn–Cr exchange is photo-inactive. The creation of heterostructures has emerged as a route towards a larger temperature range of the photomagnetic responses in certain PBAs.^92^

PBAs are relatively unique in that they can harbour both magnetism and porosity. Combining these aspects is often challenging, as the former requires short linkers that can mediate magnetic coupling and the latter larger linkers to create void space. An example of a PBA where these two properties lead to a guest-sensitive magnetic response is Co_0.41_Mn_0.59_[Cr(CN)_6_]_2/3_ [Fig. 5(b)]. Increasing humidity switches the sign of the magnetisation, driven by the water-dependent coordination environment of the Co^II^ ion, as explained above.^82^ Tetrahedral Co^II^ orders antiferromagnetically with Cr^III^, whereas octahedral coordination environments favour ferromagnetic coupling,^47^ thereby driving a change in the magnetisation. Solvent-specific effects can also be realised, *i.e.* coordination of ethanol instead of H_2_O decreases the magnetic moment and reduces *T*_c_ in the related Co[Cr(CN)_6_]_2/3_.^47^ Altogether, the saturation magnetisation, its sign, and the ordering temperatures can be susceptible to solvent effects in suitable PBA systems.

Magnetism in PBAs can also be controlled electrochemically through modification of metal oxidation states. For instance, in CrCr-based PBAs, electrochemical reduction alters both the critical temperature and coercive field: Cr^II^_1.29_Cr^III^_0.14_[Cr^III^(CN)_6_] exhibits ferrimagnetism with *T*_c_ = 240 K, while its reduced form KCr^II^_1.29_Cr^III^_0.14_[Cr^III^(CN)_6_] shows *T*_c_ = 100 K.^93,94^ Similar effects can be achieved through Li^+^ intercalation, as shown in Cr^II^_1.91_Cr^III^_0.33_[Cr^III^(CN)_6_] where sequential reduction of the Cr^III^ ions decreases *T*_c_ from 230 K to 150 K.^95^ Likewise, Li^+^ insertion in K_*x*_Cu[Fe(CN)_6_]_1−*y*_ systems changes the magnetic coupling from ferromagnetic (*T*_c_ = 20 K) to paramagnetic by reducing Fe^III^ (*S* = 1/2) to Fe^II^ (*S* = 0).^96^ The gradual nature of the electrochemical reduction enables precise control over the transition temperature,^97^ with changes largely consistent with mean-field theory. Such electrochemical control has been demonstrated in various morphologies, including powders,^95–97^ thin films,^93,98^ and core–shell particles.^99^

To briefly summarise, magnetism is well explored in PBAs and couples to an impressive range of stimuli. In principle, this is attractive for applications within *e.g.* optical switching and memory devices; yet the low ordering temperatures may hamper applications. From a practical viewpoint, the stability of *e.g.* Cr-based PBAs—which generally show the highest ordering temperatures^100^—is lower than for hexacyanoferrates or -cobaltates, which could pose a safety concern.

## 7 Strain

Abrupt lattice strains often arise in conjunction with electronic rearrangements, or may continuously appear with temperature (thermal expansion) or pressure (compressibility). From a functional viewpoint, strain can be detrimental to a particular application, but may also be exploited in devices. To illustrate, the large volume changes associated with the phase transitions in PBAs used in Na-ion batteries reduces the mechanical stability of the electrode.^101^ Conversely, strain triggered by a well-defined stimulus can allow for *e.g.* sensing of volatile organics.^102^ Understanding how volume changes couple to other functionality is thus critical.

Thermal strain (thermal expansion) is an important material consideration for devices exposed to thermal fluctuations during operation. Most conventional materials show positive thermal expansion, often with relatively small magnitudes. As an illustration, the volumetric coefficient of thermal expansion (*α*_V_) values of first-row transition metals are <70 MK^−1^.^103^ On the contrary, the expansivities of PBAs can be positive or negative (*i.e.* contraction upon heating) and *α*_V_ ranges from −120 to 150 MK^−1^.^104^ In general, the sign and magnitude of the thermal response can be explained based on the size of the metals and the free space available in the framework.^105,106^ Hence, zero thermal expansion is also accessible,^106,107^ which could be attractive in applications subject to large thermal fluctuations. In addition to continuous strain, large abrupt volume changes also result from thermally induced SCO or CT.^26,108^

Thermodynamic laws require that the overall volume of any solid is reduced when hydrostatic pressure is applied. Hence, all PBAs show negative volume strain under compression. While the counterintuitive negative thermal expansion is common in PBAs, the corresponding anomalous pressure phenomenon negative linear compressibility—expansion of one unit cell dimension under pressure—is unlikely, as it cannot occur in cubic materials. In addition to compressibility, sudden volume changes also occur from pressure-induced SCO and CT.^26,109^ Overall, the piezomechanical properties of PBAs have attracted less research attention than the thermomechanical behaviour outlined above.

**Fig. 6:**
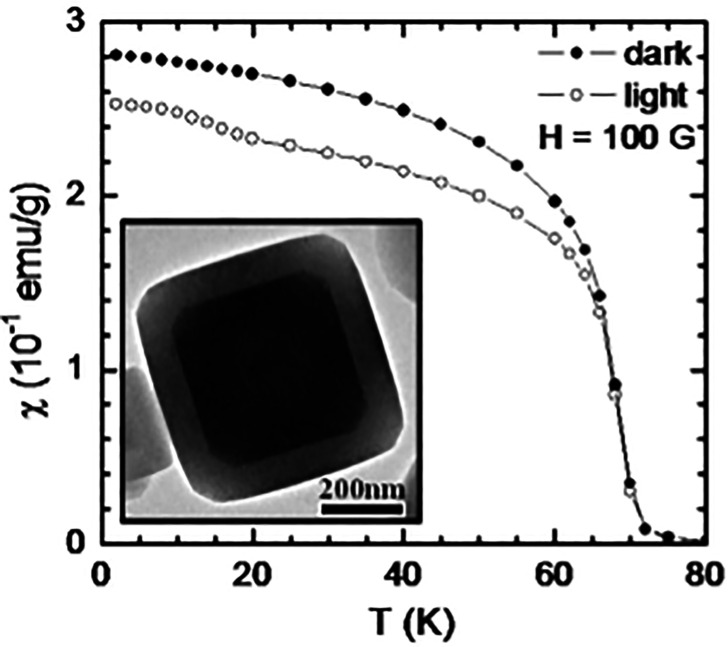
Field-cooled magnetic susceptibility of Rb*_x_*Co[Fe(CN)_6_]_1−_*_y_*/K*_x_*Ni[Cr(CN)_6_]_1−_*_y_* core–shell particles as a function of the temperature before irradiation (“dark”) and after irradiation with visible light (“light”).^111^ High-resolution transmission electron micrograph of such a particle is the inset. Adapted with permission from ref. 111. Copyright 2011 American Chemical Society.

Photoinduced charge transfer, as is well studied in A_*x*_Co[Fe(CN)_6_]_1−*y*_, is associated with a large and sudden volume change of up to 10%.^108^ This can be exploited to modify the magnetic properties in cleverly designed PBA heterostructures. By way of example, combining photoactive Rb_*x*_Co[Fe(CN)_6_]_1−*y*_ (*T*_c_ = 18 K) with photo-inactive but pressure-sensitive Rb_*x*_Ni[Cr(CN)_6_]_1−*y*_ (*T*_c_ = 70 K) allows for photo-controlled magnetism up to 70 K by means of elastic coupling between the components [Fig. 6].^92,110^ This route to extending the magnetic response window *via* strain is reported in both multilayered thin film geometries, as well as various core–shell architectures.^92,111^ X-ray induced unit cell contractions have also been demonstrated in PBAs,^25,36,112^ but the mechanism remains to be elucidated.

Porous PBAs show strains upon water uptake or release,^82^ but these effects are generally minor in comparison to the breathing transitions possible in metal–organic frameworks.^113^ An exception is the cathode material Na_2_FeFe(CN)_6_, which loses nearly 20% of its volume upon dehydration.^114^ Water strongly impacts the thermal expansion^115–117^ and compressibility,^118,119^ at least in defective samples. The possible role of solvents on the strain of SCO and CT transitions in PBAs is poorly studied, but the coupling between strain, guests, and electronic transitions is well documented in *e.g.* the related Hofmann complexes.^29^

In summary, strain unsurprisingly accompanies most electronic transitions in PBAs, such as SCO or CT, with potentially very large values. It is worth noting that the coupling between volume changes and entropy can be exploited in the emerging field of barocalorics,^120^ and good barocaloric effects were recently demonstrated in a PBA.^121^ Another highly interesting—and more well-studied—aspect of strain in PBAs is the tunable, and often negative, thermal expansion.

## 8 Opportunities and challenges

Looking forward, the research carried out to date leaves both opportunities and challenges for the field of stimuli-responsive PBAs. Table 1 summarises the currently known functionality, with stimuli and properties on the vertical and horizontal axes, respectively. As a word of caution, the stimuli-responsive behaviour of PBAs is a vast field and despite our best efforts, some accidental omissions might be inevitable. Nevertheless, Table 1 provides a useful visual guide to the applicability/susceptibility of a particular stimulus/effect. This section highlights underexplored areas in the field of stimuli-responsive PBAs and discusses avenues where we believe more research is required.

**Table 1:** Stimuli-responsive behaviour of PBAs. Stimuli and responses are shown on the vertical and horizontal axes, respectively, and tickmarks indicate where a given effect can be induced/affected by a particular stimulus. CT/SCO are used to indicate that the underlying mechanism relies on charge transfer/spin crossover

	Electric conductivity	Magnetisation	Colour	Electrochemical performance
Light		CT*	CT	✓
Temperature	✓, CT	✓, SCO, CT	SCO, CT	✓
Pressure		SCO, CT	SCO	
Magnetic field		✓		
Electrochemical	✓	✓	✓	
Water/vapour	✓	✓, CT	✓	✓
Solvents/vapours	✓		✓	✓

More examples of stimuli-responsive behaviour likely wait to be discovered in PBAs, or to be recognised as such. For example, there is little research done on field effects, particularly using electric fields, although some proof-of-concept studies are known.^32^ Moreover, PBAs with pressure-induced SCO and CT are normally piezochromic,^45^ though this descriptor is rarely used. Piezochromic framework materials are relatively uncommon, though could find applications within sensing,^122^ and hence the piezochromism in electronically active PBAs is worth noting.

Certain stimuli are also surprisingly underexplored, including the absorption of non-aqueous guest species. Most studies in this area have focused on the effect of water—if solvent effects are noted at all—whereas the solvochromic PBAs reveal that other guests may also considerably modify the properties.^53^ The importance of solvation/hydration on key properties as CT or SCO is also poorly understood, yet crucial for related compounds.^29^ Yet, one drawback of PBAs in this respect is the relatively small size of their pore windows, which limits the number of guests suitable for uptake. Nevertheless, small molecules can still penetrate and might provide another handle for property optimisation.

Another surprisingly rare property in PBAs is ferroelectricity, *i.e.* the presence of a field-switchable electronic polarisation. A few reports of ferroelectricity in PBAs are known,^123^ yet the interpretation of ferroelectric switching curves is notoriously difficult.^124^ This paucity can partially be explained by symmetry: ferroelectricity only occurs in systems adopting polar space groups. This is mutually exclusive with cubic symmetry—the most common crystal system for PBAs. Nevertheless, polarity can develop under hydrostatic compression,^118^ which shows that low-symmetry structures can be accessed under the right conditions.

**Fig. 7:**
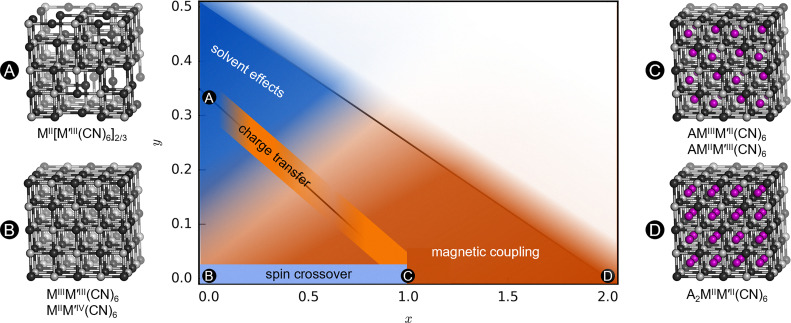
An approximate distribution of behaviour and properties in the PBA stoichiometric space (A_*x*_M[M′(CN)_6_]_1−*y*_□_*y*_·*n*H_2_O). The *x* and *y* axes refer to the number of A-site cations and vacancies, respectively, and representative crystal structures are indicated. The partially visible diagonal lines show compositions with constant oxidation state of the metals.

Like all research areas, PBAs face several challenges. For example, the molecular linker enhances the flexibility relative to oxides, but it also weakens the magnetic coupling and so most magnetic responses occur at impractically low temperatures. However, room-temperature ferrimagnetic order can be accessed by judicious choice of metals.^125^ In addition, most properties are specific to a particular stoichiometry (*e.g.* the presence or absence of vacancies); the control of which is not necessarily straightforward.^126^ Moreover, the stoichiometric ranges where a particular property is active are normally not precisely known, although exceptions exist.^127^ To aid this, Fig. 7 shows the approximate areas in the compositional space of PBAs that are occupied by different properties, to the best of our knowledge.

Moving beyond simple stoichiometry, the distribution of vacancies stands out as a largely overlooked aspect of PBA crystallography. Local-structure studies show that vacancies are not randomly distributed. Instead, they exhibit short-range correlations which can be tuned from a few to tens of nanometres by modifying growth conditions.^13,128^ Furthermore, controlling the growth direction can make the vacancy distribution anisotropic, breaking elements of the PBA′s cubic symmetry.^14^ Given this precise control, it would be valuable to explore the link between vacancy order and the various stimuli-responsive properties. For instance, the local order should directly influence the flexibility and cooperativity of the structure, which could impact the CT temperature in A_*x*_Co[Fe(CN)_6_]_1−*y*_. Moreover, since vacancies provide pores for guest molecule transport, their arrangement is likely to affect solvatochromism. Finally, the vacancy distribution might alter preferred ligand arrangements and local symmetries, thereby influencing other types of chromisms and potentially electric transport. This suggests that engineering vacancy disorder offers a powerful, yet largely unexplored, route to tailoring the stimuli-responsive properties of PBAs.

In addition to vacancies, the local order of the A-site cations and its potential influence on the properties has been largely ignored so far. For compositions AMM′(CN)_6_, a random distribution of the A-site cations leads to the space group *Fm*3̄*m* (assuming no displacive distortions), whereas alternating A order breaks the inversion symmetry and gives *F*4̄3*m*. The presence/absence of inversion symmetry is critical for certain properties, such as piezoelectricity. Yet, the conditions driving the cation (dis)order and the scope for tuning have not been studied, to date. Though similarly to the vacancies, it is likely that the cation order will be affected by the synthetic parameters. Overall, the sensitivity of the local PBA structure to the synthesis is a potential source for variation, and highlights the importance of detailed reports of experimental procedures and rigorous characterisation.

Particle size is another important design element of switchable PBAs. While photoswitching of A_*x*_Co[Fe(CN)_6_]_1−*y*_ is usually observed at intermediate values of *x* and *y*, nanosized crystalline analogues exhibit high photoswitching performance even in compositions with very small vacancy concentrations.^129^ The surrounding environment also matters: the relaxation temperature for Cs_*x*_Co[Fe(CN)_6_]_1−*y*_ differs by 55 K between particles surrounded by the cethyltrimethylammonium bromide and those embedded within the organic polymer polyvinylpyrrolidone.^129^ Likewise, Rb_*x*_Mn[Fe(CN)_6_]_1−*y*_ nanoparticles of 200 nm preserve their switching properties even in compounds with relatively low Rb content, *e.g.* Rb_0.55_Mn[Fe(CN)_6_]_0.85_.^130^ Accordingly, downsizing can expand the compositional window for electronic transitions.

The focus here has been on “traditional” PBAs with crystalline perovskite-like structures, but it is also worth highlighting more unusual topologies. An interesting recent development is amorphous PBAs.^131^ Relative to their crystalline counterparts, the amorphous systems showed improved photocatalytic properties, since amorphisation increased the number of open metal sites and modulated the bandgap.^132^ In addition to the usual cubic PBAs, a hexagonal form of Cu[Co(CN)_6_]_2/3_ with square planar CuN_4_ units and rhombohedral Zn[Co(CN)_6_]_2/3_ with tetrahedral ZnN_4_ have been demonstrated.^133,134^ Overall, the property exploration in these systems has largely focussed on absorption, and the effects of topology and crystallinity on the stimuli-responsive behaviour are still largely unknown.

## 9 Outlook

As hopefully conveyed by the previous sections, Prussian blue analogues are truly fascinating materials. Their variable stoichiometry and susceptibility to a manifold of stimuli render them highly multifunctional, often with useful commercial applications. The soft nature of PBAs also allows for modification by relatively gentle stimuli and this gives an advantage over *e.g.* oxides or metals. In addition, PBAs can harbour unusual combinations of properties—such as porosity and magnetic coupling—which opens avenues towards exciting functionality.

While PBAs as a family are extremely versatile, many properties have specific compositional and stoichiometric requirements and no single PBA displays all the functionality discussed in this perspective. Looking towards design rules, the electronic properties—spin crossover, charge transfer, and magnetic order—place obvious limits on the type of metals that can be used. Likewise, many solvent-induced responses, such as thermochromism or humidity-induced magnetic changes, require vacancies as they rely on the coordination of the solvent to the empty M-metal site. Yet the structure–property relationships are not always as obvious: for example, vacancies appear to facilitate charge transfer in A_*x*_Co[Fe(CN)_6_]_1−*y*_,^127^ but hamper the same property in A_*x*_Mn[Fe(CN)_6_]_1−*y*_.^135^ It is clear that there is still plenty to learn about the link between composition/stoichiometry and properties in PBAs.

## Conflicts of interest

There are no conflicts to declare.

## Supplementary Material

TC-013-D5TC01760B-s001

## Data Availability

No primary research results, software or code have been included and no new data were generated or analysed as part of this review.
