# Supplementary material for: Stimuli-responsive Prussian blue analogues
Source: J Mater Chem C Mater. 2025 Jul 10;13(30):15290–302. doi: 10.1039/d5tc01760b (PMC12272822; doi:10.1039/d5tc01760b)
Supplement: TC-013-D5TC01760B-s001 [file TC-013-D5TC01760B-s001.pdf]

Supplementary Information (SI) for Journal of Materials Chemistry C.
This journal is © The Royal Society of Chemistry 2025

Stimuli-responsive Prussian blue analogues

SUPPORTING INFORMATION

Hanna L. B. Boström,[a,b*] Yevheniia Kholina,[c] and Arkadiy Simonov[c*]

[a] Department of Chemistry, Stockholm University,
Svante Arrhenius väg 16C, SE-106 91 Stockholm, Sweden.

[b] Wallenberg Initiative Materials Science for Sustainability, Department of Chemistry
Stockholm University, SE-114 18 Stockholm, Sweden.

[c] Department of Materials, ETH Zürich, 8093 Zürich, Switzerland

*To whom correspondence should be addressed;
E-mail: hanna.bostrom@su.se, arkadiy.simonov@mat.ethz.ch

Table 1: Charge transfer in $A_xCo[Fe(CN)_6]_{1-y}$ as visualised in Fig. 3

A-site cation	x	y	T-switchable	$h\nu$-switchable	Ref.
-	0	0.333	no	no	1,2
Na	0.047	0.333	no	no	3
Na	0.256	0.248	yes	yes	4
Na	0.27	0.27	yes	yes	3
Na	0.308	0.231	yes		2,5
Na	0.32	0.26	yes		6
Na	0.402	0.242	yes	yes	3
Na	0.476	0.206	yes	yes	3
Na	0.5	0.175	yes	yes	7
Na	0.817	0.13	no	no	3
Na	1.077	0.231	no		2,5
K	0.025	0.325	no	no	8
K	0.12	0.3	no		9
K	0.143	0.286	no	yes	10
K	0.308	0.231	no	yes	2,5
K	0.308	0.231	yes		2,5
K	0.36	0.18	yes		9
Rb	0.45	0.175	no	yes	8
Rb	0.5	0.175	no	yes	7,11
Rb	0.52	0.16	no	yes	12
Rb	0.528	0.2	no	yes	1
Cs	0.075	0.3	no	no	13
Cs	0.175	0.275	yes	yes	13,14
Cs	0.175	0.3	yes	yes	11
Cs	0.3	0.2	yes	yes	13
Cs	0.5	0.175	no	yes	7
Cs	0.5	0.2	no		15
Cs	0.575	0.15	no	no	13
Cs	0.58	0.13	no	yes	16
Cs	0.9	0.15	no		15
Cs	0.975	0.025	no	no	8

Table 1: Charge transfer in $A_xCo[Fe(CN)_6]_{1-y}$ as visualised in Fig. 3

A-site cation	x	y	T-switchable	$h\nu$-switchable	Ref.
KCs	0.275	0.225	yes		15
KCs	0.275	0.25	yes		15
KCs	0.35	0.225	yes		15

Table 2: Charge transfer in $A_xMn[Fe(CN)_6]_{1-y}$ as visualised in Fig. 3

A-site cation	x	y	T-switchable	Ref.
K	0.7	0.1	no	17
Rb	0.38	0.18	no	18
Rb	0.58	0.14	no	19
Rb	0.59	0.14	no	20
Rb	0.609	0.13	no	21
Rb	0.716	0.099	yes	22
Rb	0.73	0.09	yes	23
Rb	0.76	0.09	yes	18
Rb	0.81	0.05	yes	20
Rb	0.82	0.04	yes	18
Rb	0.82	0.06	yes	24
Rb	0.85	0.071	yes	22
Rb	0.867	0.048	yes	25
Rb	0.88	0.04	yes	26
Rb	0.88	0.04	yes	19
Rb	0.92	0.05	yes	20
Rb	0.926	0.029	yes	22
Rb	0.94	0.02	yes	27
Rb	0.94	0.07	yes	17
Rb	0.96	0.02	yes	18
Rb	0.97	0.01	yes	26
Rb	0.97	0.01	yes	28
Rb	0.97	0.02	yes	20
Rb	0.977	0.053	yes	29

Table 2: Charge transfer in $A_xMn[Fe(CN)_6]_{1-y}$ as visualised in Fig. 3

A-site cation	x	y	T-switchable	Ref.
Rb	1	0	yes	26
Rb	1	0	yes	30
Cs	0.89	0.12	yes	17
Cs	1.51	0	yes	31

1 References

(S1) O. Sato, Y. Einaga, A. Fujishima and K. Hashimoto, *Inorg. Chem.*, 1999, **38**, 4405–4412.

(S2) T. Yokoyama, T. Ohta, O. Sato and K. Hashimoto, *Phys. Rev. B*, 1998, **58**, 8257–8266.

(S3) N. Shimamoto, S.-i. Ohkoshi, O. Sato and K. Hashimoto, *Inorg. Chem.*, 2002, **41**, 678–684.

(S4) N. Shimamoto, S. Ohkoshi, O. Sato and K. Hashimoto, *Mol. Cryst. Liq. Cryst.*, 2000, **344**, 95–100.

(S5) O. Sato, Y. Einaga, T. Iyoda, A. Fujishima and K. Hashimoto, *J. Phys. Chem. B*, 1997, **101**, 3903–3905.

(S6) S. Gawali-Salunke, F. Varret, I. Maurin, C. Enachescu, M. Malarova, K. Boukheddaden, E. Codjovi, H. Tokoro, S. Ohkoshi and K. Hashimoto, *J. Phys. Chem. B*, 2005, **109**, 8251–8256.

(S7) J.-D. Cafun, G. Champion, M.-A. Arrio, C. Cartier dit Moulin and A. Bleuzen, *J. Am. Chem. Soc.*, 2010, **132**, 11552–11559.

(S8) A. Bleuzen, C. Lomenech, V. Escax, F. Villain, F. Varret, C. Cartier dit Moulin and M. Verdaguer, *J. Am. Chem. Soc.*, 2000, **122**, 6648–6652.

(S9) H. Hanawa, Y. Moritomo, J. Tateishi, Y. Ohishi and K. Kato, *J. Phys. Soc. Jpn.*, 2004, **73**, 2759–2762.

(S10) O. Sato, T. Iyoda, A. Fujishima and K. Hashimoto, *Science*, 1996, **272**, 704–705.

(S11) J.-D. Cafun, L. Londinière, E. Rivière and A. Bleuzen, *Inorg. Chim. Acta*, 2008, **361**, 3555–3563.

(S12) A. Goujon, O. Roubeau, F. Varret, A. Dolbecq, A. Bleuzen and M. Verdaguer, *Eur. Phys. J. B*, 2000, **14**, 115–124.

(S13) V. Escax, A. Bleuzen, C. Cartier dit Moulin, F. Villain, A. Goujon, F. Varret and M. Verdaguer, *J. Am. Chem. Soc.*, 2001, **123**, 12536–12543.

(S14) A. Bleuzen, V. Escax, A. Ferrier, F. Villain, M. Verdaguer, P. Münsch and J.-P. Itié, *Angew.*

Chem. Int. Ed., 2004, **43**, 3728–3731.

(S15) M. Dronova, L. Altenschmidt, A. Bordage, E. Rivière, J.-B. Brubach, M. Verseils, G. Balthazar, P. Roy and A. Bleuzen, *Eur. J. Inorg. Chem.*, 2025, **28**, e202400443.

(S16) S. Zerdane, M. Cammarata, L. Balducci, R. Bertoni, L. Catala, S. Mazerat, T. Mallah, M. N. Pedersen, M. Wulff, K. Nakagawa, H. Tokoro, S.-i. Ohkoshi and E. Collet, *Eur. J. Inorg. Chem.*, 2018, **2018**, 272–277.

(S17) A. Regueiro, J. Castells-Gil, C. Shen, I. Mikulska, C. Allen, L. Bogani and R. Torres-Cavanillas, *Mater. Adv.*, 2024, **5**, 7473–7480.

(S18) G. Molnár, S. Cobo, T. Mahfoud, E. J. M. Vertelman, P. J. Van Koningsbruggen, P. Demont and A. Bousseksou, *J. Phys. Chem. C*, 2009, **113**, 2586–2593.

(S19) H. Tokoro, T. Matsuda, T. Nuida, Y. Moritomo, K. Ohoyama, E. D. Loutete-Dangui, K. Boukheddaden and S.-i. Ohkoshi, *Chem. Mater.*, 2008, **20**, 423–428.

(S20) E. J. M. Vertelman, E. Maccallini, D. Gournis, P. Rudolf, T. Bakas, J. Luzon, R. Broer, A. Pugzlys, T. T. A. Lummen, P. H. M. van Loosdrecht and P. J. van Koningsbruggen, *Chem. Mater.*, 2006, **18**, 1951–1963.

(S21) S. Margadonna, K. Prassides and A. N. Fitch, *Angew. Chem. Int. Ed.*, 2004, **43**, 6316–6319.

(S22) S.-i. Ohkoshi, T. Matsuda, H. Tokoro and K. Hashimoto, *Chem. Mater.*, 2005, **17**, 81–84.

(S23) H. Tokoro, T. Matsuda, S. Miyashita, K. Hashimoto and S.-i. Ohkoshi, *J. Phys. Soc. Jpn.*, 2006, **75**, 085004.

(S24) S.-i. Ohkoshi, H. Tokoro, T. Matsuda, H. Takahashi, H. Irie and K. Hashimoto, *Angew. Chem. Int. Ed.*, 2007, **46**, 3238–3241.

(S25) H. Tokoro, S.-i. Ohkoshi and K. Hashimoto, *Appl. Phys. Lett.*, 2003, **82**, 1245.

(S26) H. Tokoro, K. Hashimoto and S.-i. Ohkoshi, *J. Magn. Magn. Mater.*, 2007, **310**, 1422–1428.

(S27) A. Asahara, M. Nakajima, R. Fukaya, H. Tokoro, S.-i. Ohkoshi and T. Suemoto, *Phys. Rev. B*, 2012, **86**, 195138.

(S28) Y. Moritomo, M. Hanawa, Y. Ohishi, K. Kato, M. Takata, A. Kuriki, E. Nishibori, M. Sakata, S. Ohkoshi, H. Tokoro and K. Hashimoto, *Phys. Rev. B*, 2003, **68**, 144106.

(S29) S.-i. Ohkoshi, H. Tokoro, M. Utsunomiya, M. Mizuno, M. Abe and K. Hashimoto, *J. Phys. Chem. B*, 2002, **106**, 2423–2425.

(S30) E. J. M. Vertelman, T. T. A. Lummen, A. Meetsma, M. W. Bouwkamp, G. Molnar, P. H. M. van Loosdrecht and P. J. van Konigsbruggen, *Chem. Mater.*, 2008, **20**, 1236–1238.

(S31) T. Matsuda, H. Tokoro, K. Hashimoto and S.-i. Ohkoshi, *J. Appl. Phys.*, 2007, **101**, 09E101.
